# Delayed Post-percutaneous Nephrolithotomy Hemorrhage Managed With Superselective Renal Artery Embolization: A Nephron-Sparing Therapeutic Approach

**DOI:** 10.7759/cureus.97897

**Published:** 2025-11-26

**Authors:** Suryaram Aravind, Punith Jain R, Vivek Meyyappan, Velmurugan Palaniyandi, Hariharasudhan Sekar, Sriram Krishnamoorthy

**Affiliations:** 1 Urology, Sri Ramachandra Institute of Higher Education and Research, Chennai, IND; 2 Urology and Renal Transplantation, Sri Ramachandra Institute of Higher Education and Research, Chennai, IND

**Keywords:** calculi, embolization, hemorrhage, kidney, percutaneous nephrolithotomy

## Abstract

Percutaneous nephrolithotomy (PCNL) is the preferred treatment for large renal stones, though delayed arterial bleeding is a rare but serious complication. A 59-year-old male, previously discharged post-PCNL, presented on postoperative day 10 with gross hematuria and clot retention. CT angiography confirmed an interpolar segmental artery pseudoaneurysm. Superselective renal artery embolization (SSAE) with n-butyl cyanoacrylate achieved hemostasis without transfusion. Renal function was preserved, and the patient recovered uneventfully. The patient was reviewed after two weeks for follow-up after SSAE. This case illustrates the value of prompt vascular imaging in delayed post-PCNL bleeding and highlights the role of superselective renal artery angioembolization as a safe, effective, nephron-sparing intervention in modern endourological practice.

## Introduction

Percutaneous nephrolithotomy (PCNL) has become the gold standard for managing large and complex renal calculi, including staghorn stones and multiple calyceal stones [1,2]. Its global acceptance lies in its high stone clearance rates and minimally invasive nature compared with open or laparoscopic alternatives. Despite its efficacy, hemorrhagic complications remain the most feared and adverse, leading to significant morbidity and, rarely, mortality. Minor bleeding is common in the immediate postoperative period and usually resolves with conservative measures, but severe or delayed hemorrhage presents a distinct clinical challenge requiring urgent evaluation and intervention. Bleeding following PCNL can be multifactorial. Vascular injury may occur during various steps of the procedure, including puncture and tract dilation, guidewire manipulation, stone fragmentation, or excessive nephroscopic torque [3]. Post-PCNL hemorrhage has been reported in approximately 0.3-1% of cases requiring angioembolization, although minor bleeding is seen in up to 10-15% of patients. While venous bleeding is typically self-limiting, arterial trauma can lead to vascular lesions such as pseudoaneurysms or arteriovenous fistulae. These lesions may manifest as recurrent hematuria, clot retention, flank pain, or hemodynamic instability days to weeks after the index procedure.

Measures such as hydration, bladder irrigation, blood transfusion, and temporary nephrostomy clamping may suffice in minor cases as a conservative management protocol. However, if hematuria persists, if there is a drop in hemoglobin greater than 2 g/dL, or if the patient experiences hemodynamic instability, further imaging is necessary. Contrast-enhanced CT (CECT) reliably identifies vascular injuries, while digital subtraction angiography (DSA) remains the gold standard, providing both diagnostic and therapeutic interventions [4].

The introduction of superselective angioembolization (SSAE) has transformed the management of post-procedural complications following PCNL [5]. Superselective catheterization of the bleeding vessel allows precise occlusion while preserving uninvolved renal parenchyma in SSAE. The nephron-sparing approach is essential for patients with solitary kidneys, bilateral disease, or pre-existing renal impairment. Success rates surpass 90% in most series. SSAE has become the first-line intervention for effectively managing significant post-PCNL hemorrhage, thereby minimizing the risk of nephrectomy while ensuring long-term preservation of renal function [6,7].

This article highlights the role of SSAE in managing delayed post-PCNL hemorrhage through a representative case. It discusses its clinical implications, outcomes, and long-term relevance in preserving renal function while safeguarding patient safety.

## Case presentation

**Figure 1:**
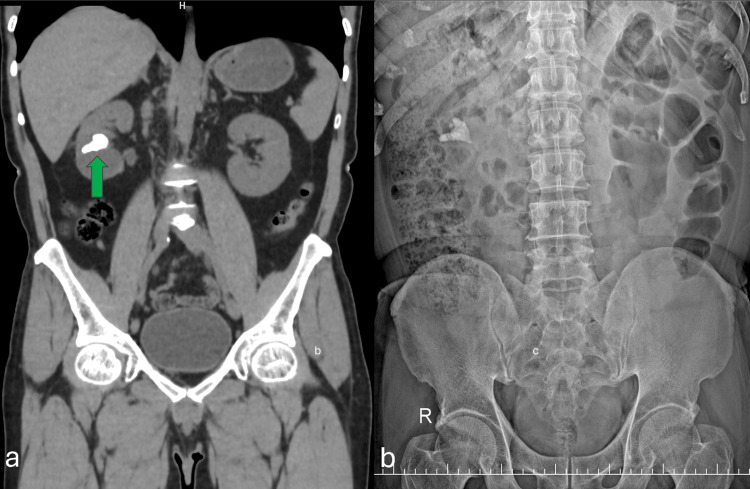
Coronal CT and plain abdominal radiograph demonstrating urinary tract calculi. (a) Coronal non-contrast CT of the abdomen reveals a large radiodense calculus within the left renal pelvis (green arrow). (b) Corresponding plain abdominal radiograph (kidney, ureter, bladder) shows radiopaque shadows consistent with urinary calculi in the left kidney.

A 59-year-old male with a right partial staghorn calculus (Figure 1) and normal baseline renal function was scheduled for PCNL. He had no significant comorbidities. After preoperative evaluation, PCNL was performed in the prone position via a single lower calyceal puncture under fluoroscopic guidance using the triangulation technique. The tract was dilated, and stone clearance was achieved without intraoperative complications. Hemostasis was confirmed, a double-J stent was placed, and a nephrostomy tube was secured. The patient recovered uneventfully and was discharged on the second postoperative day with clear urine output.

Ten days later, he developed sudden-onset gross hematuria with clot retention, leading to difficulty in voiding and lower abdominal pain. On readmission, he was pale but hemodynamically stable. Hemoglobin dropped to 8 g/dL (baseline hemoglobin: 12.1 g/dL). Non-contrast CT of the kidney, ureter, and bladder revealed multiple bladder clots, for which the patient underwent cystoscopy with clot evacuation under local anesthesia. Supportive measures, including hydration, antibiotics, bladder irrigation, and serial hemoglobin monitoring, were initiated. Even so, hematuria continued, and hemoglobin levels kept going down.

**Figure 2:**
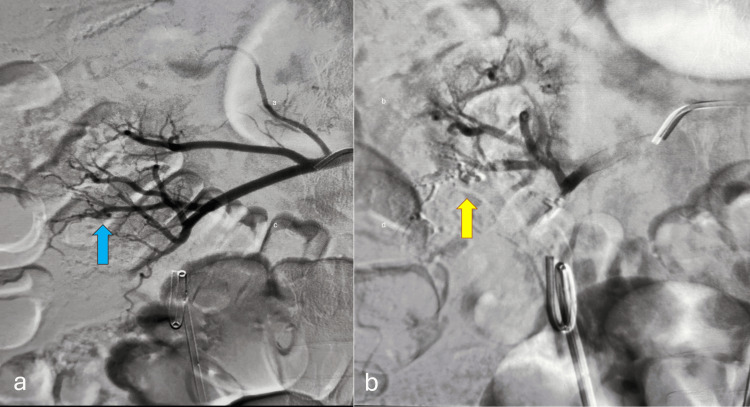
Selective renal angiography demonstrating vascular anatomy and embolization of a renal pseudoaneurysm. (a) Digital subtraction angiography of the left renal artery shows a well-opacified pseudoaneurysm arising from a segmental branch (blue arrow). (b) Post-embolization angiogram following selective coil deployment demonstrates complete occlusion of the pseudoaneurysm sac with cessation of abnormal contrast opacification (yellow arrow), while preserving perfusion of the uninvolved renal parenchyma.

**Figure 3:**
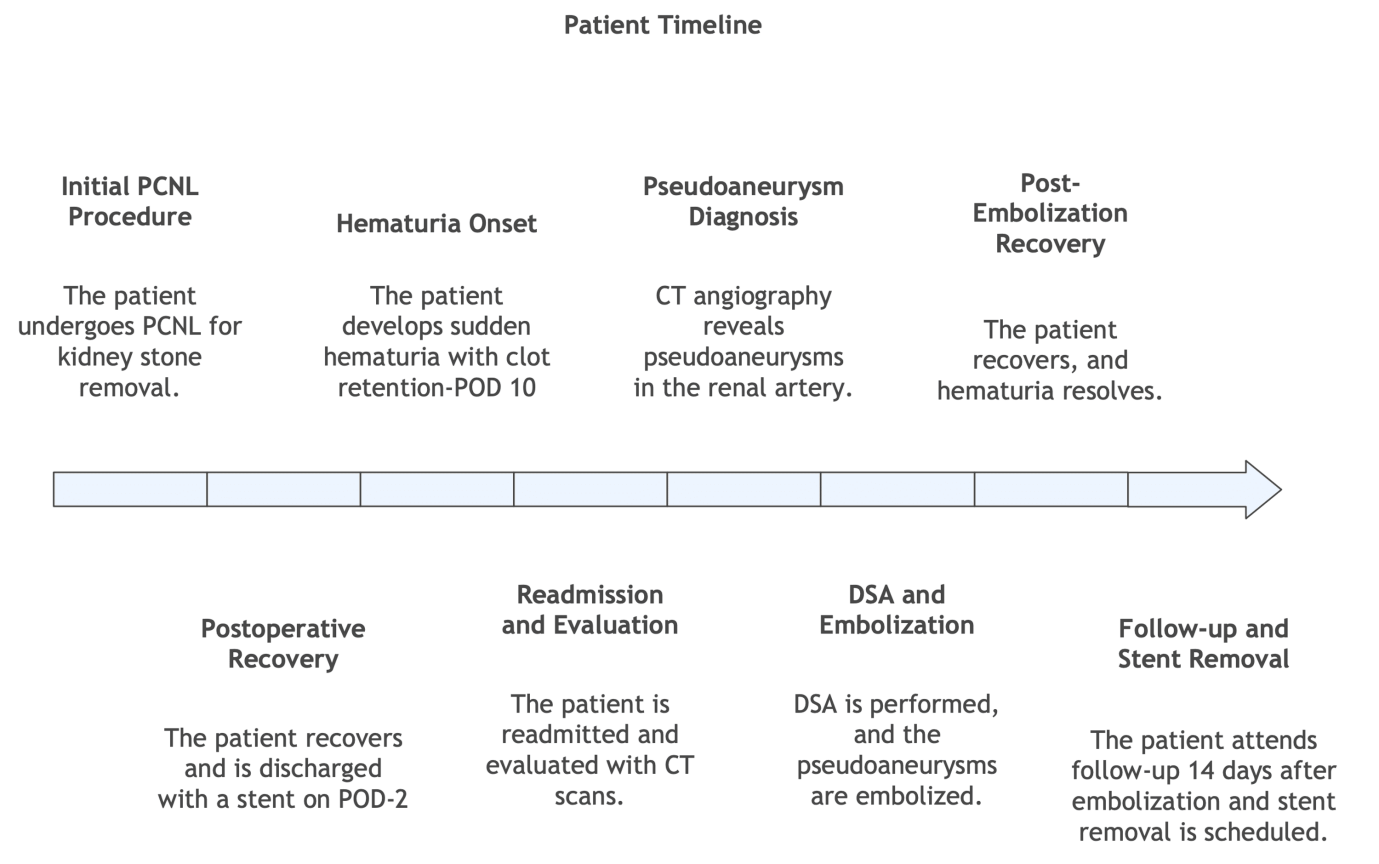
Patient’s treatment timeline. DSA = digital subtraction angiography; PCNL = percutaneous nephrolithotomy; POD = postoperative day

CT angiography showed small pseudoaneurysms coming from the segmental renal artery of the anterior division of the interpolar artery. This is in line with delayed arterial injury after PCNL (Figure 2). After a multidisciplinary discussion, DSA was performed. The bleeder vessel was selectively catheterized, and embolization was achieved using 25% n-butyl cyanoacrylate (NBCA) glue. The embolic agent was delivered in a controlled manner to occlude the pseudoaneurysm while preserving adjacent parenchymal perfusion. Post-embolization angiography confirmed successful occlusion without contrast extravasation. The patient tolerated the procedure well, and his urine gradually cleared. Hematuria resolved, and hemoglobin levels stabilized without requiring a transfusion. He was discharged on the second day after embolization, ambulant and stable. At the two-week follow-up, he remained asymptomatic, with preserved renal function: serum creatinine, 1.0 mg/dL; estimated glomerular filtration rate, 95 mL/minute/1.73 m²; and hemoglobin, 11.3 g/dL. There has been no recurrence of hematuria. Since the patient had SSAE and a double-J stent in situ, he was given a course of oral antibiotics. Double-J stent removal was scheduled for four weeks. A timeline of the patient’s hospital course and treatment is presented in Figure 3.

## Discussion

Hemorrhage following PCNL is a well-documented but relatively infrequent complication. Significant bleeding requiring intervention occurs in fewer than 5% of cases. Most episodes are intraoperative and resolve with tamponade or nephrostomy clamping. Unlike immediate bleeding, which can often be venous and self-limiting, delayed hemorrhage, as observed in this case, typically originates from arterial injury and necessitates specialized evaluation and management [8]. This delayed presentation often stems from vascular trauma to segmental or interlobar arteries during the PCNL procedure, either during tract creation or stone manipulation. Such injury to the intimal lining of the vessel can lead to the formation of pseudoaneurysms or arteriovenous fistulas [9,10]. These vascular lesions frequently remain asymptomatic for several days, only becoming clinically evident when factors such as clot lysis or increased intraluminal pressure trigger their rupture. Recognizing this specific timeline is crucial, as delayed bleeding commonly manifests after patients have been discharged from the hospital, emphasizing the value of educating patients and ensuring prompt reporting of any hematuria they experience [11].

Patients experiencing delayed bleeding after PCNL may present with gross hematuria, retention of clots, flank pain, anemia, or, in severe instances, signs of hemodynamic instability. Clinical evaluation becomes necessary when hematuria is associated with a decrease in hemoglobin of more than 2 g/dL or if conservative treatments do not control the bleeding. CECT angiography serves as the preferred diagnostic tool, offering rapid detection of pseudoaneurysms, arteriovenous fistulas, or active bleeding sites [12]. DSA remains the definitive gold standard, allowing for precise localization of the vascular lesion and enabling immediate therapeutic embolization to control hemorrhage efficiently.

Post-PCNL vascular injury management guidelines have undergone significant evolution. Historically, uncontrolled bleeding often required surgical exploration or nephrectomy, resulting in substantial morbidity and parenchymal loss. SSAE now offers a minimally invasive, nephron-sparing alternative. Reported technical success rates exceed 90%, and clinical success, defined as cessation of hematuria without recurrence, for clarity, in the vast majority of patients. The choice of embolic material is guided by the type of lesion and the operator’s preference [13].

Coils provide permanent occlusion, ideal for discrete pseudoaneurysms. Table 1 shows how different embolic agents compare to each other. Polyvinyl alcohol particles and Gelfoam are temporary substances that are often used to stop diffuse oozing. NBCA glue, as used in this case, is particularly effective for high-flow or technically challenging lesions due to its rapid polymerization and durable occlusion properties. While each agent possesses unique strengths and risks, the use of superselective techniques maximizes renal preservation. Complications of SSAE are infrequent but may encompass access-site hematomas, contrast-induced nephropathy (1-3%), and segmental infarction due to non-target embolization. These risks can be minimized through careful technique. Post-embolization syndrome, i.e., flank pain and fever, is usually self-limiting. Significantly, long-term renal function is maintained in the majority of patients, even following bilateral embolization, thereby underscoring the safety of this method [14].

**Table 1:** Comparison of various embolic agents. AVF = arteriovenous fistula; NBCA = N-butyl cyanoacrylate; PVA = polyvinyl alcoho

Agent	Type	Mechanism/Key feature	Strengths	Limitations/Best use
Coils	Permanent	Induce thrombosis at the target site	Precise and effective for pseudoaneurysms	Limited for diffuse bleeding; best for discrete lesions
PVA particles	Temporary, semi-permanent	Block small arterioles	Widely available; low cost; suitable for diffuse oozing	Risk of non-target embolization; possible recanalization
NBCA glue	Permanent	Polymerizes rapidly on contact with blood	Excellent for high-flow lesions; durable occlusion	Needs expertise; risk of non-target spread
Onyx	Permanent	Slow, controlled liquid deposition	Precise, controlled delivery; good for complex AVFs	Expensive; limited availability

The present case demonstrates the value of SSAE in bridging the gap between conservative management and radical surgery. Despite persistent hematuria and hemoglobin decline, nephron preservation was achieved through superselective embolization with NBCA glue, which ensured durable occlusion of the pseudoaneurysm while avoiding nephrectomy. Recent studies provide robust support for this paradigm. Large multicenter studies have demonstrated the efficacy and safety of SSAE for post-PCNL vascular injuries, establishing it as the standard of care for refractory or delayed bleeding. Long-term outcomes further indicate minimal impact on renal function, affirming its nephron-sparing advantage [15]. In the broader context of endourology, this case illustrates the value of multidisciplinary collaboration. Urologists must stay vigilant for delayed hemorrhage, while interventional radiologists are crucial for rapid diagnosis and treatment. Timely recognition, rapid imaging, and prompt intervention ensure optimal outcomes, minimize morbidity, and preserve renal function.

Take-home messages

PCNL is generally effective and safe; however, a risk of delayed bleeding complications remains, which may develop after hospital discharge. Recognizing this risk and promptly investigating a trauma evaluation of hematuria with imaging are essential for timely management. SSAE has revolutionized the management, offering a minimally invasive, nephron-preserving option that yields excellent outcomes with low complication rates. Urologists should have a low threshold for ordering vascular imaging in patients with unexplained hematuria or a decline in hemoglobin after PCNL, and close coordination with interventional radiology is critical. By adopting this proactive approach, clinicians can ensure prompt intervention, minimize morbidity, and preserve renal function. Ultimately, SSAE is no longer a last resort therapy but an integral part of modern endourological care for managing post-PCNL hemorrhage.

## Conclusions

SSAE is a highly effective, safe, and minimally invasive intervention for addressing delayed hemorrhage post-PCNL. It offers an alternative to open surgery by specifically targeting the vessel, thereby preserving renal function. With higher technical success rates and minimal long-term renal impairment, SSAE has become the treatment of choice in this clinical setting. Early detection of vascular injuries, facilitated by CT angiography and confirmed by DSA, allows precise localization of the bleeding source and timely therapeutic embolization. The diverse availability of embolic materials, ranging from coils and polyvinyl alcohol particles to NBCA glue, enables tailored therapy. This report highlights the importance of incorporating SSAE into the management algorithm for PCNL-related bleeding and underscores the value of multidisciplinary collaboration between urology and interventional radiology teams.
